# GPs’ suspicion of child abuse: how does it arise and what is the follow-up?

**DOI:** 10.1080/02813432.2020.1755784

**Published:** 2020-04-24

**Authors:** Erik Stolper, Jan Paul Verdenius, Geert-Jan Dinant, Margje van de Wiel

**Affiliations:** a CAPHRI School for Public Health and Primary Care, University of Maastricht, Maastricht, The Netherlands;; b Faculty of Medicine and Health Sciences, Department of Primary and Interdisciplinary Care, University of Antwerp, Antwerp, Belgium;; c General practice, Heeg, the Netherlands;; d Department of Work and Social Psychology, Faculty of Psychology and Neuroscience, Maastricht University, Maastricht, The Netherlands

**Keywords:** Suspicion of child abuse, child maltreatment, general practitioner, family practice, gut feelings, diagnostic methods, barriers in management

## Abstract

**Background:** Child abuse is widespread, occurs in all cultures and communities, remains undiscovered in 90% of cases and has serious long-term effects. Physicians generally underidentify and underreport child abuse. To understand this low reporting rate and how the suspicion of child abuse arises, we examined GPs’ experiences.

**Research questions:** How does the suspicion of child abuse arise in GPs’ diagnostic reasoning? How do they act upon their suspicion and which barriers do they encounter in their management?

**Methods:** Twenty-six GPs participated in four focus groups. We used purposive sampling to include GPs with different levels of experience. We performed a thematic content analysis.

**Results:** Suspicion of child abuse arose from common triggers and a gut feeling that ‘something is wrong here’. GPs acted upon their suspicion by gathering more data, through history taking and physical examination. They often found it difficult to decide whether a child was abused, because parents, despite good intentions, may simply lack parenting skills and have different values. Clear signs of sexual abuse and physical violence were institutionally reported by GPs, whereas in less clear-cut cases they followed them up and built a supporting network of professionals around the family.

**Conclusions:** A low child abuse reporting rate by GPs to CACRC does not mean a low detection rate. In trying to improve a child’s situation, GPs make use of patients’ trust in their doctor by involving other professionals. Awareness of the role of gut feelings in developing a suspicion may increase early detection and preventive actions.

**Key points**
- Physicians generally underidentify and underreport child abuse.
- Suspicion of child abuse arose from common triggers and a gut feeling that ‘something is wrong here’.
- GPs acted upon their suspicion by gathering more data, through history taking and physical examination.
- GPs found it difficult to decide whether a child was abused, because parents, despite good intentions, may lack parenting skills.

## Introduction

Child abuse is a wide spread phenomenon, occurs in every culture and community, remains undiscovered in 90% of the cases and has serious long-term effects [1–4]. Physicians generally underidentify and underreport child abuse [5]. Worldwide, rates of child abuse are more than twice as high in low- and middle-income sectors of society, and cause more fatalities [1,6]. Child abuse cases mostly concern emotional and physical neglect and emotional maltreatment [1,7]. A minority (21%) also concerns physical maltreatment and sexual abuse [7]. In nearly all cases, a biological parent is the perpetrator (96%) [5]. In the Netherlands, it affects about 90,000–127,000 children (0–17 years old; 3% of all children) resulting in at least 17 child fatalities a year [7]. Much research has been done into the identification and management of child abuse, resulting in risk assessment tools, lists of triggering signs and symptoms and protocols [8–11].

General practitioners (GPs) are key figures in health care organisations and often know a lot about the context of their patients, such as their family and socio-economic situation and medical history. They often see children and one would expect that they would regularly note child abuse. However, reports from developed countries show that reporting rates of child abuse by GPs to a Child Abuse Counselling and Reporting Centre (CACRC) are low [12,13]. The reasons for this low reporting rate have been studied among GPs, e.g. in the US, Australia, France and Sweden [5,8,14]. Uncertainty about the diagnosis, a fear of harming the relationship with parents, low confidence in CACRCs, lack of knowledge about risk factors and pressure of time were important reasons mentioned. Instead of reporting, GPs sometimes referred suspected cases to other health care providers or monitored them by regular follow-ups during office hours [14]. Also Dutch GPs did not often approach a CACRC for advice (2,7%) or to report child abuse (1,6%). A retrospective study found that GPs working in an out-of-hours GP Service often failed to recognise child abuse [15]. Dutch police, schoolteachers and hospital specialists more often reported child abuse to a CACRC (33,9%, 4,8% and 8,5%, respectively) [16].

There is a lack of studies examining how the suspicion of child abuse arises in general practice and how the diagnosis is established. Of course, in clear cases of child abuse bruises, fractures and neglect are evident signals. However, child abuse is often more hidden, and the signals are vague. Diagnostic reasoning is generally a mixture of analytical and non-analytical, intuitive aspects, where gut feelings help GPs to navigate safely in uncertain situations [17–19]. Previous research among GPs in Europe showed that they acknowledge gut feelings a valuable diagnostic tool [17,19,20]. We expect that gut feelings contribute to the development of a suspicion of child abuse. Therefore, we studied how the suspicion of child abuse arises in GPs, how they act upon this suspicion, and how they deal with identified abuse. Additionally, we wanted to know what causes the low reporting rate by Dutch GPs. For these reasons we examined GPs’ views of, and experiences with child abuse.

## Research questions

How does the suspicion of child abuse arise in GPs’ diagnostic reasoning? How do GPs act upon their suspicion and which barriers do they encounter in their management?

## Methods

Twenty-six GPs (16 females) participated in four focus groups. We used purposive sampling to include GPs with different levels of experience (mean 16 years, range 1–35 years), working in rural (*n* = 5) and urban areas, and in communities with different socio-economic levels, spread across the Netherlands. We invited them to discuss the topic of ‘vulnerable children’, without disclosing the exact purpose of the focus groups. The sessions were led by experienced moderators using an interview guide addressing the research questions. Data saturation was reached after four sessions. All sessions were audio-recorded and transcribed. In the thematic content analysis, two authors (ES and MW) coded the emerging subthemes in the data based on the main research themes, using the data management tool NVivo [21,22]. They developed a code book during an initial interactive coding process and both coded all data till agreement was reached. Almost all codes occurred in every focus group. Patterns and emergent trends in the data were analysed. Quotes illustrating the results are provided below with focus group number and participant number between brackets. The results section is structured along the main coding categories: development of a suspicion, acting upon a suspicion, and reflection.

## Results

GPs in all groups talked about child abuse cases (*N* = 47). They also told about cases they had missed (*N* = 9), unjustified suspicions (*N* = 4) and vulnerable children (*N* = 10).

## Development of a suspicion

### Triggers

Many triggers were mentioned, such as a young child with cystitis or vaginal mycosis, a child***’***s unexpected behaviour change, a single parent situation, malnutrition, school absenteeism and bruises at unusual parts of the body*. I think the girl first came in for cystitis, and when we asked her there was also something like vaginal discharge or redness, that sort of thing. And then the girl also said something about it herself…something strange about her mother****’****s boyfriend. So, you immediately thought:* ***‘****friend of her mother****’****s, what****’****s that about?****’*** *[FG3,7]*

### The role of gut feelings

All groups discussed the gut feeling described as there is something wrong here and considered it a valuable diagnostic tool. In such cases, GPs got an uneasy feeling, while listening to complaints or observing a child, which put them on the alert. An odd symptom or unusual behaviour, such as a child behaving like *‘an unguided missile in my office’ [FG4,1]* and the intimidating reaction of its parents might make GPs think there is something wrong. If they had a gut feeling, most GPs tried to objectify it. *What I also find suspicious is when, for instance, children immediately sit on my lap. That makes me think, like, hey, this is unusual, a child that doesn’t know me at all. That sort of makes me uncomfortable. It kind of gives me a sense of is this child really safe? [FG4,3] If I really get a sense of alarm, the kind where I think I’ll be worrying about this tonight, then I take action the very same day. Cos I don’t want to lie in bed at night thinking might this child be in danger.[FG2,3]*

### Child abuse or a lack of parenting skills?

Participants sometimes struggled with the question whether it was a case of child abuse or a lack of parenting skills. They recognised that children’s development might be endangered when the quality of parenting was low in their view, but hesitated to call it child abuse. They found it hard to discuss this with parents and tried to avoid the term child abuse as it might hinder the communication. *I usually see people failing even though they really mean well, and still there’s a threat to the child.[FG4,1] In whatever way you express [child abuse], to parents, it’s hardly a message that you can give in such a way.[FG4,6]*

### Frame of reference

In all focus groups, participants discussed the problem that there is no uniform frame of reference to judge the quality of parenting. The judgment is based on their own personal and culturally determined view of what is good parenting. *Of course, there are many families where you think, well, you know, their values are different from mine. Does that warrant a report? I don’t think so.[FG3,8]*

### Contextual knowledge

GPs’ contextual knowledge was an important background against which they weighed up their observations and findings. Locum GPs mentioned that they lacked this information*. And we do know some families, err, you’re extra vigilant with these very complex families where there really are problems.[FG3,2]*

## Acting upon a suspicion

### Starting a discussion

GPs often tried to discuss their worries with the parents. GPs approached parents in their own personal style, some being more confrontational than others. *When we think something’s wrong, we should try and discuss that suspicion with people to create an opening. But if you do so in a rather direct, confrontational way, that might put an immediate stop to the relationship, and you lose sight of them.[FG1,1] I’m rather outspoken. With those kind of people I would tell them in clear terms, what you’re doing just isn’t right for your little one.[FG2,4]*

### Shifting boundaries

GPs weighed arguments for or against certain actions. They wondered whether they had enough arguments to report a suspicion to CACRC. They sometimes hesitated whether to report to CACRC or give the parents another opportunity to improve the child’s situation. *I do see they’re working on it, and that the situation is gradually improving, but it’s so slow that I wonder if it’s wise to wait.[FG2,3] In such a situation there’s a great risk -at least that’s what I think- that not setting strict boundaries leads to shifting boundaries.[FG2,4]*

### Further diagnostics

When GPs were in doubt about child abuse, they initiated further diagnostics, first by history taking and examination or checking the patient record. Some GPs visited patients at home or urged them to come to the office for regular check-ups. They might also refer the child to a paediatrician or other professional to gain new information and perspectives for further diagnostics, as this did not interfere with their relationship with the parents. *Then took a look at the file and it turned out she had also had other vaginal symptoms as she had fallen onto the armrest of a chair with her legs spread.[FG4,6] Paediatricians know all about that, just rule out such and such somatic problems and then they go and see a child psychologist. That means less of a burden [than if we as GPs address the problem] [FG3,2.]*

### Support

GPs aimed to improve children’s situation. People tend to trust their GP, which enables GPs to motivate parents to accept educational or social support. This is often available nearby, in their own family, and there are easily accessible professionals such as practice nurses, social workers connected to schools, youth health physicians and nurses and educationalists. *They [these professionals] have a much broader perspective, looking at whatever else is the matter.[FG4,3] And the threshold for parents is much lower.[FG3,8] If you maintain a relationship, you know an entrance, can motivate the parents to go and see the practice-nurse, specialised in youth health care, who has more time, can discuss issues, and has all kinds of options, for instance contacting the school.[FG3,3]*

## Documentation

GPs generally documented their suspicions in the patient records, usually avoiding the term child abuse when describing the situation. Some locum GPs passed on their suspicions to the regular GP using a separate note. Sometimes GPs used a pop-up to alert them for the next encounter. *When I was standing in for a GP on holiday, I wrote it down on the transfer form: this is what struck me in this family.[FG2,1]*

## Reporting to CACRC

Participants rarely reported child abuse to CACRC. Occasionally they told parents that they considered reporting the situation to CACRC. They did involve CACRC in cases of sexual abuse and physical violence. They valued the possibility of asking CACRC for advice without mentioning the name of the patient, but the procedure was not always clear. *So I actually made one report in 28 years.[FG3,1] We’ve now muddled through for long enough. You’ll have to do better or else I’ll have to report you.[FG2,3]* Some participants doubted the effectiveness of CACRC, as the institution did not always act the way the GPs had expected them to. Some GPs reported disappointing experiences with CACRC or negative consequences, such as parents changing doctors after they had involved CACRC. Some mentioned that parents often have a negative opinion of CACRC. *The two reports I have filed really got me into trouble with the parents … Cos they were immediately branded as child abusers, without further investigation… And the mother was really furious then… She filed a complaint.[FG2,3] I don’t really have a lot of faith in CACRC.[FG4,2]* Not reporting to CACRC did not mean that GPs did not act upon their suspicion. *You’re investing a lot of effort in it, but you didn’t report it to the agency. Well, lots of GPs have reported very little to the agency, but they often have to deal with child abuse.[FG4,6]*

## Reflection

### Learning

The discussion in the focus groups as such was a learning experience for the participants, as they learned from each other’s perspectives and approaches. They became aware of shortcomings in their knowledge about how CACRC works; they learned from their colleagues’ solutions in difficult situations, such as involving educational experts; they reflected on their lack of attentiveness to child abuse and their need for more expertise. *If you’re in doubt about a child’s development, just visit their home, that really tells you a lot more.[FG4,1] Indeed I don’t really do that often enough.[FG4,4] Perhaps I should do that more often too, just always ask them to fully undress [for examination].[FG2,1]*

### Barriers to diagnosing child abuse

GPs mentioned that they often lacked the time and relevant information, e.g. from the children’s school, to take the broader context of a child’s situation into account, sometimes because they were biased by having known the family for a long time. GPs then might tend to ignore a gut feeling or to disregard certain problems. *When you get a call from CACRC, you actually very often find you know nothing at all about the child, that it’s been absent from school a lot.[FG3,7] How often are you confronted with those kinds of feelings like, there’s something wrong here, and, er, yes, perhaps one of our characteristics is the tendency to suppress that feeling.[FG1,1] The longer you’ve known a family, the less objective you become, by definition. Because obviously you develop a bond with people.[FG3,3]*.

### Missed cases

Participants in two groups reported a total of 9 missed cases. The GPs involved regretted that they had not recognised the child abuse, but, in hindsight, they were not able to point out how they could have recognised it. In most cases, patients (i.e. the children) had not dared to talk. The GPs learned from these experiences to always think through the problems patients presented: what’s going on here? *The mother was physically disabled, and it was only years later that I found out the girl was being abused by her father. So then I think: goodness, I never noticed that.[FG3,2]*

### Differences related to experience

Recently qualified GPs were more likely to consider child abuse and to consult the reporting code, also because they had less contextual knowledge. *So I think that younger doctors are relatively more objective.[FG3,3] I think you have an added value as a locum GP, you have a different perspective.[FG2,5]*

## Discussion

The focus group sessions showed that GPs’ suspicions of child abuse were based on various triggers. The gut feeling that something is wrong here played an important alerting role. GPs acted upon their suspicions by gathering more data by means of history taking and physical examination. They often hesitated to call it child abuse, as it might be a case of parents lacking parenting skills, despite their good intentions. GPs struggled with the values regarding good parenting. Whereas clear signs of sexual abuse and physical violence were reported by GPs to CACRC, in cases of emotional child abuse they tended to follow the case up and build a supporting network of professionals around the family. Most GPs highly valued the patient–doctor relationship but recognised the risk of shifting boundaries at the expense of the child. A low child abuse reporting rate by GPs to CACRC did not imply a low detection rate. GPs used their patients’ trust in them to improve a child’s situation by involving other professionals, rather than reporting to CACRC.

This study was the first one in the Netherlands to ask GPs themselves about their diagnostic reasoning regarding child abuse. The qualitative method of focus groups resulted in rich data, gathered from participants all over the Netherlands, with different levels of expertise and working in communities with different socio-economic levels. The GPs accepted our invitation to discuss their experiences with vulnerable children and were unaware of our focus on child abuse, ensuring high external validity of the study.

Research into barriers of child abuse management in other countries has showed similar findings, such as uncertainty about the suspicion, prioritising a good relationship with parents, lack of confidence in organisations like CACRC, and lack of time [5,14,23].

This study clearly shows how GPs focussed their attention on the very first step in the diagnostic reasoning process of recognising child abuse, i.e. becoming aware of a sense of alarm that there might be something wrong with the child. This uneasy feeling arises automatically, based on triggers in the observation, history taking and examination of the child, and it induces GPs to look for the often still hidden triggers, by more careful observation, questioning and examination, e.g. asking the child to undress. When the signs of child abuse are very clear, a gut feeling does not contribute much to the correct diagnosis. It is, however, in the large grey area of obscure child abuse that this first step, becoming aware of a sense of alarm, might be most important in detecting child abuse, without which a GP is unlikely to start checking the reporting code or a list of risk factors. In general practice, physicians are often faced with uncertain and complex situations, and gut feelings steer them like a compass [17,18], evidently also in the domain of child abuse. As the cognitive processes in diagnostic reasoning are generic, we think that GPs awareness of and further research into the role of gut feelings in developing a suspicion of child abuse and acting upon it may help to improve the situation of children at risk.

Another prominent finding was the lack of a gold standard for the diagnosis of child abuse. In medicine, physicians are used to comparing working hypotheses with a standard reference, a well-described disease with its signs and symptoms and typical course of development [24], but in the case of emotional maltreatment, the GPs’ frame of reference is less clear, as judgment depends on values determined by personal and cultural views. This judgmental aspect of diagnosing and reporting child abuse proved to be a real struggle for many of the GPs in this study.

These difficulties of judgement GPs experienced are similar to those in the domain of public health concerning the introduction and implementation of the national Child Index [25]. The aim of this index is to timely detect children at risk. But when is a child at risk? The intended reporters, such as teachers and youth health physicians, struggle with dilemma’s comparable to those of GPs. Our study suggests that GPs do recognise possible cases of child abuse more often than they report them to CACRC. In contrast to other professionals who more frequently report child abuse, such as the police, schoolteachers and paediatricians, GPs maintain a long-standing relationship of care with their patients, based on mutual trust. They are well familiar with the context of the families and their histories, which appears to be both an advantage and a disadvantage. The close relationship enables GPs to build a network of supporting professionals around the family and the child. The same close relationship, however, might prevent GPs from recognising child abuse, or mean they refrain too long from taking the necessary action. In cases of emotional maltreatments, which constitute the majority of child abuse cases, they may act upon the problem themselves, or may involve easily accessible professionals whom they know and trust. When the desired results are not achieved as well as in clear-cut cases such as physical violence or sexual abuse, GPs do report to CACRC. Not reporting to CACRC may offer GPs and other professionals opportunities to improve the child’s situation themselves. Nevertheless, this is not without its risks, for instance because the follow-up of an abuse situation might be inadequate when parents do not comply with agreed check-ups during office hours, which may not be noticed by the GP. Moreover, GPs often do not have a comprehensive overview of all experts involved and the effects of their interventions. In contrast with CACRC, GPs mostly do not receive information from other sources such as the school, neighbours or the police. Additionally, GPs probably see only the tip of the iceberg, as people involved in child abuse might avoid seeing their GP.

GPs’ views about the role of CACRC is sometimes based on disappointing experiences with this agency, causing distrust, lack of knowledge about the formal procedures and unjustified expectations. Some GPs were afraid to lose control of what will happen when involving CACRC. This lack of trust in organisations like CACRC by GPs has been found in other countries as well [5,14,26].

The more experienced GPs are, the less they report to CACRC, and the less frequently they participate in continuing education on child abuse [14,27]. In our study, we found indications that young GPs tend to consider child abuse, and use the reporting code, at an earlier stage than older GPs do. This might be due to more attention being focussed on the topic in current GP training, and their often more objective view of the child’s situation.

Our study showed that sharing child abuse cases in small groups was a learning experience in itself. In addition to general postgraduate courses on the topic, CACRC might offer education in small groups where GPs learn from each other and from an CACRC expert [15].

Finally, as regards the situation in the Netherlands, we were surprised to discover a gap between the available knowledge about child abuse provided in articles, reports, postgraduate courses, guidelines and collaboration agreements [9,28,29], and the considerable lack of knowledge about the way GPs detect and manage child abuse in everyday practice. The current top–down approach seems to assume that the more information an authority offers, the better field workers will act and the more surely targets will be met. Knowing the determinants, such as a list of risk factors, and how to act upon them, is apparently thought to be sufficient [30]. The recent, more regulatory step taken by the Dutch government, which oblige professionals to mandatorily discuss even suspicions of child abuse with CACRC, is probably based on the same assumption. In Sweden, mandatorily reporting turned out to be not very successful in primary care [14]. More research into this issue is needed.

In conclusion, GPs are concerned about identifying and managing child abuse, despite their low reporting rate to CACRC. The situation of abused children might be improved by greater attention for the crucial role of gut feelings in raising suspicion, and for ways to act upon these feelings, as well as for a better collaboration between GPs and CACRC, providing GPs with support that fits their work style.
